# Current status of healthcare financial literacy among medical trainees and junior hospitalists: An observational survey study

**DOI:** 10.1097/MD.0000000000041581

**Published:** 2025-02-14

**Authors:** Odera Ekeh, Amonte Simmons, Alka Farmer, Krystal Hunter, Lin Zheng

**Affiliations:** a Cooper Medical School of Rowan University, Camden, NJ; b Division of Hospital Medicine, Department of Medicine, Cooper Medical School of Rowan University, Camden, NJ; c Department of Medicine, Inspira Health Network, Vineland, NJ.

**Keywords:** healthcare financial literacy, healthcare outcomes, hospitalists, medical education, medical students.

## Abstract

The absence of comprehensive education on hospital value-based purchasing during undergraduate medical education may lead to inadequate comprehension of healthcare finance during Graduate Medical Education and subsequent medical practice. Medicine residents and junior hospitalists (defined as those who completed their residency in the last 5 years) may lack essential healthcare financial skills to address the needs of their patients and the healthcare system effectively. To assess healthcare financial literacy among medical students, medicine residents, and junior hospitalists, we conducted a survey at an academic Internal Medicine residency affiliated with a US Medical School. Participants completed a 40-item questionnaire sourced from federal government healthcare system websites, providing demographic data and indicating prior formal healthcare finance education. Of the 126 respondents, only 15.6% reported receiving formal healthcare financial education, with merely 4 out of 34 junior hospitalists having prior education. Notably, there were no significant differences in correct answer percentages between 3rd and 4th-year medical students, medicine residents, and junior hospitalists across 3 domains: Medicaid/Medicare insurance, US healthcare systems & insurance, and healthcare access, quality and value-based purchasing. However, participants performed better in the domain of Medicaid/Medicare insurance compared to the other domains. This study underscores the potential deficiency in financial skills among junior healthcare providers, highlighting the importance of addressing this gap to ensure effective patient care.

## 1. Introduction

The landscape of the healthcare system in the United States (US) has undergone significant transformation in the past decade, particularly with the implementation of the Affordable Care Act in 2010. This legislation aimed to expand healthcare coverage to more US citizens and shift the reimbursement methodology from a fee-for-service model to 1 centered on value-based reimbursement. A notable component of these changes is the introduction of hospital value-based purchasing by the Centers for Medicare & Medicaid Services. HVBP seeks to enhance the hospital payment system by incorporating healthcare quality data. Its overarching goal is to foster improved clinical outcomes and enhance patient experience within the acute care setting.^[1]^

Against this backdrop of change, the concept of health literacy emerges as a critical factor in shaping healthcare delivery, especially among hospitalists. Health literacy is defined as the degree to which individuals have the capacity to obtain, process, and understand basic health information and services needed to make appropriate health decisions.^[2]^ Low health literacy in patients is associated with poorer health outcomes and poorer use of health care services.^[3]^ For healthcare providers, better healthcare financial literacy is pivotal to understanding the innovative framework of HVBP in redesigning care delivery to achieve better outcomes for patients and reduce costs. However, there are only a limited number of medical schools in the country that have incorporated HVBP education into their curricula to emphasize the skills necessary to maximize value to patients.^[4]^ As a result, the status of healthcare finance in the 3rd- and 4th-year medical students, internal medicine residents, and junior hospitalist physicians (defined as who completed their residency in the last 5 years), herein referred to as junior healthcare providers, is largely unknown. There is a scarcity of studies examining the level of knowledge among medical students and junior healthcare providers regarding the current state of the US healthcare system. Agrawal et al conducted a study to assess medical students’ understanding of the US Health Care System. Among 770 respondents, nearly all were aware of the adverse consequences of lacking insurance; however, 27% were unaware that the US has the highest healthcare expenses compared to other nations.^[5]^ Similarly, Simon et al,^[6]^ observed a negative perception among medical students and residents regarding managed care in the US, underscoring a deficiency in specific knowledge among junior healthcare providers. Furthermore, a recent study conducted in Italy revealed significant knowledge gaps among resident physicians concerning healthcare organization and management.^[7]^

The lack of comprehensive education on value-based purchasing (VBP) in undergraduate medical education may result in a deficient understanding of healthcare finance during graduate medical education and later in medical practice. Moreover, this deficiency could potentially have adverse effects on patient outcomes. While it might be assumed that residency training and clinical practice expose medicine residents and junior hospitalists to HVBP, there is currently no research confirming this assumption. Therefore, this prospective pilot survey study aims to assess the level of healthcare system financial literacy among junior healthcare providers. We sought to characterize the healthcare financial literacy of medical students, medicine residents and Hospitalists at an academic Internal Medicine residency facilitated with a US Medical School.

## 2. Materials and methods

This study was approved by our institutional review board (IRB). The study consists of a voluntary questionnaire sent to 3rd and 4th-year medical students, internal medicine resident physicians and junior Hospitalists at Cooper Medical School of Rowan University and Cooper Hospital in Camden, NJ. The voluntary questionnaire was administered over a 6-month period from May 2023 to November 2023. Survey responses were recorded anonymously using Microsoft 360 forms. The questionnaire consisted of 40 knowledge-based questions derived from federal government healthcare system websites and databases. Questions had only 1 correct answer and overall were divided into 3 sets of knowledge-based question domains that included: Medicaid/Medicare insurance; US healthcare systems and healthcare insurance; and healthcare access, quality, and value-based purchasing.

### 2.1. Participants

Inclusion criteria induced medical students in either 3rd or 4th year, medicine resident physicians in internal medicine serving clinical responsibilities at Cooper University Hospital, and junior hospitalists in the division of hospital medicine at Cooper Health System.

### 2.2. Data analysis

Comparisons between healthcare financial knowledge scores among the 3rd/4th-year medical students, medicine residents, and junior hospitalist were run using 1-way ANOVA testing with Tukey post hoc testing. The post hoc test was run to run pairwise comparisons between each group. All analysis was complete using SPSS (IBM, Armonk).

## 3. Results

A sum of 126 respondents participated in the survey. Among them, 46% (58 respondents) were medical students, while 54% (68 respondents) were internal medicine residents and junior hospitalists. A sum of 60% of the respondents identified as female, while 30% identified as male. A total of 15.6% of all respondents reported having received some formal healthcare financial education. Of the 34 medicine residents surveyed, 8 (23.5%) indicated they had received some formal healthcare financial education previously. In contrast, only 4 out of 34 junior hospitalists reported having received such an education before (Table 1).

**Table 1:** The characteristics of survey respondents, stratified by the level of training.

Training level	Medical students		Medicine residents		Junior hospitalist	
	M3	31	PGY1	18		34
	M4	27	PGY2&3	16		
Total		58		34		34
Sex	Male	15		20		15
	Female	43		14		19
Prior healthcare financial training		8 (13.8%)		8 (23.5%)		4 (11.8%)

**Figure 1.**
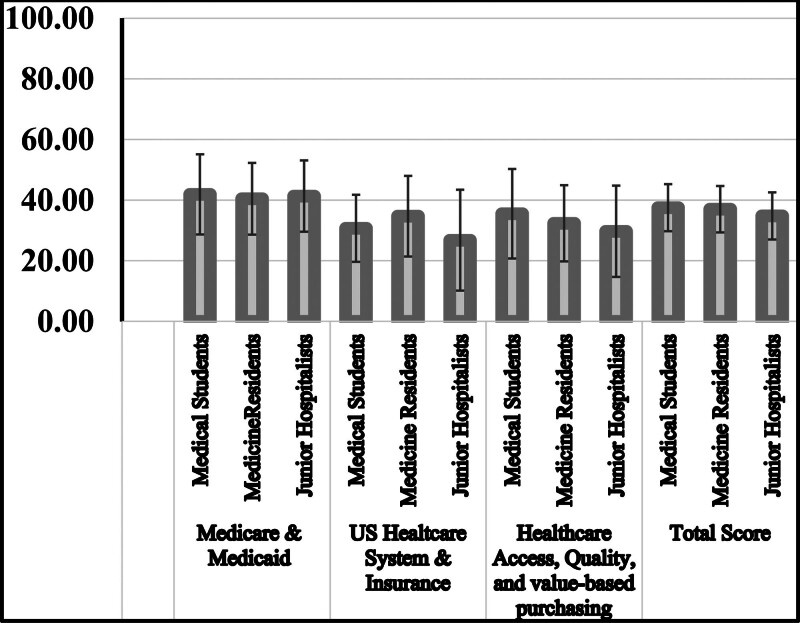
The average number of questions answered correctly on healthcare financial literacy test, stratified by the topic and level of training.

There were no significant differences in the mean percentage of correct answers to questions between 3rd and 4th-year medical students, medicine residents, and junior hospitalists (Table 2). In the Medicaid & Medicare domain, medical students scored a mean percentage of 41.90% (±13.84%), medicine resident scored 40.44% (±11.83%) while junior hospitalists scored 41.32% (±11.76%). There is no statistical significance between medical students and medicine residents (*P* = .852), medical students and junior hospitalists (*P* = .975), and between medicine residents and junior hospitalists (*P* = .954; Tables 2 and 3). In the US healthcare system and healthcare insurance domain, medical students achieved a mean percentage of 30.69% (±11.06%), compared to a mean percentage of 34.71% (±13.31%) in medicine resident physicians, and mean percentage of 26.76% (±16.65%) in junior hospitalists. There were no significant differences among groups (medical students vs medicine residents, *P* = .348 nor medical students vs junior hospitalists, *P* = .365) for this domain. However, there was a significant difference between medicine residents versus junior hospitalists (*P* = .041). Regarding the healthcare access, quality, and value-based purchasing questions, medical students scored a mean percentage of 35.52% (±14.77%), medicine resident physicians scored 32.35% (±12.57%). Whereas junior hospitalists scored 29.71% (±15.07%). There are no significant differences among the groups (medical students vs medicine residents, *P* = .563, medical students vs junior hospitalists, *P* = .148, medicine residents vs junior hospitalists, *P* = .726). Overall, the mean percentage of correct answers for medical students and medicine residents were 37.50% (±7.75%) and 36.99 (±7.66%) respectively. Junior hospitalists had an average score of 34.78% (±7.77%). Likewise, there is no statistical significance in overall performance between each group (medical students vs medicine residents [*P* = .949], medical students vs junior hospitalists [*P* = .237], medicine residents vs junior hospitalists [*P* = .469]; Tables 2 and 3). Moreover, we also found these conclusions among mean percentages were not influenced by “survey respondents’ sex” or having previous financial education (data not shown). Overall, survey participants performed better in the domain of Medicaid/Medicare insurance compared to the domains of US healthcare systems and insurance, healthcare access, quality, and value-based purchasing (Fig. 1).

**Table 2:** Responders’ correct response rate in each healthcare financial literacy domain.

		N	Mean (%)	Standard deviation	*P* value
Medicare & Medicaid	Medical students	58	41.90	13.24	.865
	Medicine residents	34	40.44	11.83	
	Junior hospitalists	34	41.32	11.76	
US healthcare system and insurance	Medical students	58	30.69	11.06	.053
	Medicine residents	34	34.71	13.31	
	Junior hospitalists	34	26.76	16.65	
Healthcare access, quality, and value-based purchasing	Medical students	58	35.52	14.77	.164
	Medicine residents	34	32.35	12.57	
	Junior hospitalists	34	29.71	15.07	
Total score	Medical students	58	37.50	7.75	.256
	Medicine residents	34	36.99	7.66	
	Junior hospitalists	34	34.78	7.77	

**Table 3:** T test results.

Medicare & Medicaid	Medical students	Medicine residents	Junior hospitalists
Medical students		.852	.975
Medicine residents	.852		.954
Junior hospitalists	.975	.954	

US healthcare system and insurance			
Medical students		.348	.365
Medicine residents	.348		.041
Junior hospitalists	.365	.041	

Healthcare access, quality, and value-based purchasing			
Medical students		.563	.148
Medicine residents	.563		.726
Attending	.148	.726	

Total score			
Medical students		.949	.237
Residents	.949		.469
Junior hospitalists	.237	.469	

*P* values showed no statistical significance between medical students, medicine residents, and junior hospitalists in the domain of Medicaid/Medicare insurance, US healthcare systems and healthcare insurance, and healthcare access, quality, and value-based purchasing.

## 4. Discussion

There is a notable lack of understanding of healthcare financial literacy among physicians and medical students. Limited prior studies evaluating healthcare financial literacy have found that resident physicians struggle to seize high-value, cost-conscious care learning opportunities in the hospital setting.^[8]^ To enhance the delivery of high-value, cost-conscious care and address the escalating healthcare expenditures, hospitals have increasingly relied on hospitalists.^[9]^ Hospital medicine has emerged as one of the rapidly growing specialties, with a national count of over 44,000 hospitalists.^[10]^ Additionally, a study analyzing data from surveys conducted by the Association of American Medical Colleges on medical school graduates from 2018 to 2020 found that 19% of respondents expressed an interest in pursuing a career as a hospitalist.^[11]^ However, to our knowledge, no study has specifically investigated the healthcare financial literacy of hospitalists. Despite conducting searches using relevant keywords such as “Hospitalists, physicians, healthcare financial literacy,” no research on this subject was identified in PubMed. While existing research primarily focuses on physicians’ personal financial literacy,^[12,13]^ it is crucial to recognize that a lack of healthcare finance literacy among healthcare providers could have adverse effects on care delivery and result in the inefficient use of valuable healthcare resources.

Our study revealed that only 12% of respondents, including junior hospitalists, reported receiving any formal healthcare financial education. Efforts to address this issue have been made by educational leaders and regulators, who have focused on developing competency frameworks for individual physicians. For example, the Accreditation Council for Graduate Medical Education has integrated patient safety, quality improvement, care transitions, high-value care, and population health into its systems-based practice core competency.^[14]^ The objective of these initiatives is to equip physicians with the skills to deliver patient-centered, high-quality care while remaining mindful of costs. Despite these efforts, resistance to change has hindered significant progress.^[15–17]^ Graduate medical education programs have faced challenges teaching and assessing these newer competencies, so even recent physician graduates may lack the full complement of skills necessary to address patient and health care system needs.

The limitations of healthcare finance literacy training during undergraduate medical education and residency have resulted in residents struggling to seize high-value, cost-conscious care learning opportunities in the workplace setting.^[8]^ Consequently, many of today’s academic faculty were not trained in these competencies. In our study, only 4 out 34 junior hospitalists indicated they received formal healthcare financial education before. This might explain why medicine residents scored better than junior hospitalists in the domain of US healthcare system and healthcare insurance (*P* = .041, Table 3). While it might be assumed that residency training and clinical practice expose medical residents and junior providers to hospital value-based purchasing (HVBP), our study found that clinical exposure alone does not necessarily lead to a better understanding of healthcare financial literacy. Across all healthcare literacy domains covered in our survey, namely Medicaid/Medicare insurance, US healthcare systems and insurance, healthcare access, quality, and value-based purchasing, junior hospitalists did not demonstrate superiority when compared to medicine residents and 3rd/4th-year medical students (Tables 2 and 3). To some extent, medical students performed slightly better in all 3 domains, which could be attributed to the recent introduction of healthcare financial education into our medical school’s curricula.

The potential deficit in healthcare finance literacy among healthcare providers may disproportionately impact certain communities, with insured patients faring better than those who are not. Consequently, the Affordable Care Act significantly increased the number of insured patients, particularly those from low-income communities. As a result of this shift, many patients in lower-income areas rely on Medicaid/Medicare rather than private insurance. Recent research indicates that patients covered by Medicaid/Medicare, while insured, receive lower-quality care from physicians compared to those with private insurance.^[18]^ The disparity in quality-of-care stems from various factors, including low reimbursement rates and high administrative burdens, which hinder physicians’ ability to provide optimal care. However, beyond these challenges, a lack of healthcare finance literacy among providers may also have a detrimental impact on patient care. Insufficient understanding of health finance among physicians can pose obstacles when treating patients covered by Medicaid/Medicare. It’s crucial for our medical students and residents to actively engage in developing interprofessional relationships. We should educate trainees on effective collaboration with social workers, dietitians, physical therapists, advanced practitioners, and administrative leaders within our clinical and hospital systems.

This pilot survey study was conducted at a single institution with a limited number of participants, potentially limiting its generalizability. Recruitment for the survey was voluntary, and participation among hospitalists was somewhat restricted, potentially introducing selection bias. The investigators formulated twenty questions in Medicare & Medicaid, and ten questions in the domains of US healthcare systems and healthcare insurance, and healthcare access, quality &value-based purchasing. The responses to these questions may not fully reflect the healthcare literacy of the participants in each domain due to sample representativeness.

## 5. Conclusions

In essence, mastery of the language of business and finance appears to coincide with enhanced patient outcomes. Our research has pinpointed shortcomings in healthcare financial literacy among junior healthcare providers. This study indicates that junior healthcare providers may lack crucial financial skills necessary to effectively meet the needs of their patients and navigate the healthcare system. It appears that current residency training and clinical practice may not sufficiently address this literacy gap. Such inadequacies could disproportionately impact vulnerable communities, particularly those covered by Medicare/Medicaid. To address this issue effectively, collaboration among medical schools, residency programs, and hospitalist programs is imperative in bridging this gap.

## Acknowledgments

We thank the section editor and all reviewers of this paper for their critical and thorough comments.

## Author contributions

**Conceptualization:** Odera Ekeh, Alka Farmer, Lin Zheng.

**Data curation:** Odera Ekeh, Lin Zheng.

**Formal analysis:** Krystal Hunter.

**Investigation:** Odera Ekeh, Amonte Simmons, Lin Zheng.

**Methodology:** Odera Ekeh, Amonte Simmons, Lin Zheng.

**Project administration:** Odera Ekeh, Lin Zheng.

**Supervision:** Lin Zheng.

**Validation:** Lin Zheng.

**Writing – original draft:** Lin Zheng.

**Writing – review & editing:** Odera Ekeh, Amonte Simmons, Krystal Hunter, Alka Farmer, Lin Zheng.
